# Long-Term Clinical Protection from Falciparum Malaria Is Strongly Associated with IgG3 Antibodies to Merozoite Surface Protein 3

**DOI:** 10.1371/journal.pmed.0040320

**Published:** 2007-11-13

**Authors:** Christian Roussilhon, Claude Oeuvray, Christine Müller-Graf, Adama Tall, Christophe Rogier, Jean-François Trape, Michael Theisen, Aissatou Balde, Jean-Louis Pérignon, Pierre Druilhe

**Affiliations:** 1 Bio-Medical Parasitology Unit, Institut Pasteur, Paris, France; 2 Laboratoire d'Ecologie Parasitaire Evolutive, Pierre et Marie Curie Université, Paris, France; 3 Unité d'Épidémiologie, Institut Pasteur de Dakar, Senegal; 4 Institut de Médecine Tropicale, Service de Santé des Armées, Marseille, France; 5 UR 077 Paludologie Afro-Tropicale, Institut de Recherche pour le Développement, Dakar, Sénégal; 6 Statenseruminstitut, Copenhagen, Denmark; 7 Unite d'Immunologie Parasitaire, Institut Pasteur de Dakar, Senegal; University of London, United Kingdom

## Abstract

**Background:**

Surrogate markers of protective immunity to malaria in humans are needed to rationalize malaria vaccine discovery and development. In an effort to identify such markers, and thereby provide a clue to the complex equation malaria vaccine development is facing, we investigated the relationship between protection acquired through exposure in the field with naturally occurring immune responses (i.e., induced by the parasite) to molecules that are considered as valuable vaccine candidates.

**Methods and Findings:**

We analyzed, under comparative conditions, the antibody responses of each of six isotypes to five leading malaria vaccine candidates in relation to protection acquired by exposure to natural challenges in 217 of the 247 inhabitants of the African village of Dielmo, Senegal (96 children and 121 older adolescents and adults). The status of susceptibility or resistance to malaria was determined by active case detection performed daily by medical doctors over 6 y from a unique follow**-**up study of this village. Of the 30 immune responses measured, only one, antibodies of the IgG3 isotype directed to merozoite surface protein 3 (MSP3), was strongly associated with clinical protection against malaria in all age groups, i.e., independently of age. This immunological parameter had a higher statistical significance than the sickle cell trait, the strongest factor of protection known against Plasmodium falciparum. A single determination of antibody was significantly associated with the clinical outcome over six consecutive years in children submitted to massive natural parasite challenges by mosquitoes (over three parasite inoculations per week). Finally, the target epitopes of these antibodies were found to be fully conserved.

**Conclusions:**

Since anti-MSP3 IgG3 antibodies can naturally develop along with protection against P. falciparum infection in young children, our results provide the encouraging indication that these antibodies should be possible to elicit by vaccination early in life. Since these antibodies have been found to achieve parasite killing under in vitro and in vivo conditions, and since they can be readily elicited by immunisation in naïve volunteers, our immunoepidemiological findings support the further development of MSP3-based vaccine formulations.

## Abstract 2

Using data from malaria cases in Senegal, Pierre Druilhe and colleagues found that antibodies of the IgG3 isotype directed to merozoite surface protein 3 (MSP3) were strongly predictive of clinical outcome.

## Editors' Summary

**Background.:**

Malaria kills about one million people—mainly children—every year. Most of these deaths are caused by Plasmodium falciparum, a parasite transmitted to people through the bites of infected mosquitoes. In the human body, the parasites replicate in liver cells before changing into so-called “merozoites.” These infect red blood cells, where they replicate rapidly before bursting out and infecting more red blood cells. This massive increase in the number of parasites in the body causes a fever and can also damage vital organs. Although individuals can protect themselves against being bitten by mosquitoes, a vaccine is urgently needed to reduce the global burden of malaria. Vaccines help the immune system fight infectious diseases. When a disease-causing organism (pathogen) enters the human body, the immune system produces antibodies. These are proteins that recognize molecules (antigens) on the pathogen and that enlist other parts of the immune system to kill the invader. This process is often slow the first time around, so people can be ill for a while. However, the immune system “remembers” the experience and responds much quicker to subsequent attacks by the same pathogen. Vaccines, which contain antigens from pathogens, prepare the immune system so that it responds quickly and effectively to a pathogen's first attack.

**Why Was This Study Done?:**

An effective vaccine against merozoites would limit the severity of malaria and prevent many deaths, but scientists do not know which of the antigens on merozoites stimulate a protective immune response. Each candidate therefore has to be tested in long, expensive field trials. Young children who live where malaria is endemic (i.e., always present) have frequent attacks of malaria but gradually develop natural immunity to the disease so that after the age 10 y, even though there are always parasites in their blood, they rarely become ill. If researchers knew which malaria antigens and which parts of the immune response are involved in natural immunity, they could use this information in vaccine design. In this study, the researchers have investigated which type of antibody (antibodies come in different varieties or “isotypes,” each of which does a slightly different job in the immune system) and which of five potential malaria antigens is associated with protection against clinical malaria.

**What Did the Researchers Do and Find?:**

In 1990, the researchers started a unique study in Dielmo, Senegal, West Africa, a small village where malaria is transmission is very high all year round. For 7 y (although the initial analysis concentrated on data from the first two years) medical staff visited every villager daily to look for clinical signs of malaria. Diagnoses of malaria were checked by looking for parasites in blood samples. One year into the study, the researchers tested blood from each participant for antibodies that recognized the candidate malaria antigens. When they analyzed their data, they found that only one immune response—the production of antibodies of the IgG3 isotype directed against an antigen called merozoite surface protein 3 (MSP3)—was strongly associated with clinical protection against malaria in all age groups. The researchers also found that some of the children developed protection against malaria when they were very young. Furthermore, in children, production of anti-MSP3 IgG3 in 1991 was associated with protection against malaria for the next 6 y.

**What Do These Findings Mean?:**

These findings show that the production of IgG3 antibodies that recognize MSP3 is strongly associated with the development of long-lasting natural protection against malaria. They also show that both protection and these antibodies can develop in very young children. Malaria transmission is unusually high in Dielmo, so these findings may not necessarily be relevant to regions where transmission is lower. In addition, although other research has shown that antibodies of this type can kill parasites in test tubes and in animals, it is possible that these antibodies are markers of, rather than causes of, protective immunity against malaria. Nevertheless, these findings support the continued development of MSP3-based vaccines, particularly since they suggest that early vaccination has the potential to protect infants against life-threatening attacks of malaria.

**Additional Information.:**

Please access these Web sites via the online version of this summary at http://dx.doi.org/10.1371/journal.pmed.0040320.

- The MedlinePlus encyclopedia contains pages on malaria and on vaccination (in English and Spanish)
- Information is available from the World Health Organization on malaria (in English, Spanish, French, Russian, Arabic, and Chinese)
- The US Centers for Disease Control and Prevention provide information on malaria and on immune responses to malaria (in English and Spanish)
- Information is available from the Malaria Vaccine Initiative on malaria and malaria vaccine development

## Introduction

A malaria vaccine is urgently needed [1,2]; however, the rational development of such a vaccine has suffered from a lack of knowledge of the relevance of experimental models [3]. This has resulted in a highly unsatisfactory situation in which each hypothesis derived from such models is investigated in lengthy and costly clinical trials. The lack of a reliable surrogate marker of protection in humans is thus a recognized limitation to the identification and development of efficacious vaccines [4].

The role of antibodies in clinical protection against malaria erythrocytic stages has long been recognized by in vivo transfer of antibodies from protected African adults to nonprotected individuals infected with P. falciparum [5,6]. However, several decades later it remains unclear which of the many antibody specificities contained in such sera may play a critical role, and hence which of the corresponding antigen(s) may represent potential vaccine candidate(s). Identification of such antigens requires the characterization of antibody species in relation to the precisely defined medical status of individuals exposed under field conditions, and in a longitudinal manner, since malaria attacks are spread over time. The corresponding epidemiological conditions were therefore established in an area of Senegal so as to fulfil two main features: (i) to include active case detection, i.e., by daily medical visits to each individual over several years and by providing access to medical care 24 h a day, (ii) making use of improved diagnostic criteria in order to distinguish malaria from other fevers, namely the pyrogenic threshold of parasite density as defined in this particular area [7], the validity of which was confirmed independently [8,9]. Employing these criteria represents a substantial improvement in the assessment of bona fide clinical malaria episodes since, as shown below, both refractory and susceptible individuals can be accurately identified using these criteria in all age groups.

Most immunoclinical studies have dealt with a single antigen in a given location, precluding any comparative assessment of the relevance of each antigen. The aim of the present study was to correlate clinical protection in an endemic population with the immune response to five leading malarial vaccine candidates that are currently underway in, or about to enter, numerous clinical trials (see list of trials at http://www.who.int/vaccine_research/documents/en/malaria_table.pdf) [1]. Four of these molecules are the targets of antibodies that inhibit red blood cell invasion, namely merozoite surface protein 1 (MSP1) [10], MSP2 [11], apical membrane antigen 1 (AMA1) [12,13], and ring-infected erythrocyte surface antigen (RESA) [14], whereas MSP3 is targeted by cytophilic antibodies inhibiting intra-erythrocytic parasite growth in a monocyte-dependent manner [15].

## Methods

### Study Area and Collection of Clinical Data

The village of Dielmo (13°45′N, 16°25′W) is localized in one of the rare areas of Senegal, West Africa where malaria is holoendemic (experiencing perennially a high level of transmission by mosquitoes, due to the presence of a permanent stream), with an average of 5.16 infective bites per week during the first 2 y of the survey [16]. The 247 inhabitants of Dielmo village were enrolled in a prospective study using to our knowledge one of the most stringent protocols of clinical follow-up ever applied in the field and consisting of daily surveillance by medical staff (present 24 h/d, 7 d/wk) in order to identify and to analyse all episodes of morbidity [16]. The field set-up was designed and tested over 1 y before the actual study was conducted (e.g., questionnaires used for daily surveillance were written in three languages, and the reliability of responses were systematically addressed).

Each villager was visited daily at home and had the ability to consult at any time one of the two medical doctors permanently on-site. In the event of a report or complaint of fever, headache, or vomiting, a medical examination and three thick blood films were made. One of the thick blood smears was Giemsa-stained and examined immediately on-site for the purpose of deciding on treatment. The other two slides were dehaemoglobinized, stained, and examined in our central laboratory in Dakar, using more rigorous and standardized conditions with quality control assessment [16]. The results of the latter slides were used for the present study.

The criteria leading to a given episode of morbidity being attributed to malaria have been studied in detail and defined previously [7]: a malaria attack was defined as an episode of fever (temperature >38.5 °C) associated with a parasite density exceeding an age-dependent pyrogenic threshold described for this village (the parasite density threshold for each age group was determined to be 24,500 parasites/μl at ages < 12 mo; 27,000 at 12–23 mo; 24,000 at 2 y; 20,000 at 5 y; 15,500 at 10 y; 10,000 at 20 y; 7,500 at 30 y; and 5,000 in adults older than 40 y) [7]. The improved ability of this criterion to distinguish malaria attacks from other causes of fever has been documented [17] and has been further confirmed independently in different African settings [18–20].

Antimalarial treatment was initiated in all participants with confirmed malaria attack according to previously established criteria [7,21]. During the study period, treatment relied on the administration of quinine chlorhydrate (Quinimax), chosen in view of its efficacy in this area of chloroquine resistance and its fast effect, given orally at a dose of 8 mg/kg under medical supervision (with assessment of proper ingestion) at 8 h intervals (i.e., 25 mg/kg/d) for 7 d. It is of note that the very short half-life of quinine has the advantage that it avoids a buildup of a prophylactic concentration of the drug in the receivers' blood that could provide artificial protection in the following weeks or months, i.e., the influence of this confounding factor is avoided. Since free medical care was available 24 h/d, self-treatment was most uncommon, as was ascertained by systematic detection of antimalarial drugs previously [16].

All villagers were farmers with equivalent economic and social status. Entomological studies were conducted 15 d/mo, year-round, by 12 investigators rotating each night from one household to another. These studies were conducted for several years simultaneously with clinical data recording. They did not show any substantial differences in exposure to *Anopheles* from one house to the other, and clinical cases were at similar prevalence in all households. The average entomological inoculation rates reported in this paper correspond to the actual entomological inoculation rates measured during the same years of the clinical survey.

The Dielmo Project was initiated in 1990, and the present study initially focussed on the period from July 1990 to July 1992; over that two-year period we calculated that each individual received an average of 520 *P. falciparum-*infected bites. The serum samples were collected in a cross-sectional study in May and June 1991. The clinical data collected from July 1990 to July 1991 were used for retrospective statistical analysis, and the clinical data collected between July 1991 and July 1992 were used for prospective statistical studies. Since the village continued to be followed up clinically according to the same criteria, and in view of results obtained from July 1990 to July 1992, clinical data obtained over the ensuing 5 y (up to July 1997) were employed for the long-term analysis.

### Ethical Approval

The informed consent of each villager (or that of the parents in the case of children) was obtained at the beginning of the study after a thorough explanation of its purpose and was renewed at the beginning of each year of the survey. The study design received clearance from the Senegal National Ethics Committee (Dakar, Senegal).

### Antigens

The peptide MSP3-b used in this study is part of the C-terminal domain of the MSP3 DG210 protein and has been described previously [22,23]. It is located in the highly conserved C-terminal region of the molecule (amino acids 185–254) [24,25] (Oeuvray et al., unpublished data). It defines a B cell epitope targeted by naturally occurring antibodies. Human affinity-purified antibodies on peptide MSP3-b react with the parasite native protein in Western blots of P. falciparum schizont extracts, and in immunofluorescence on infected RBCs, thus showing the relevance of the peptide epitope to the original protein, and inhibit parasite growth in cooperation with blood monocytes, in vitro or in vivo, in passive transfer experiments [24,25]. The RESA peptide was purchased from Bachem (Bubendorf, Switzerland). The same sera were tested under the same experimental conditions using the MSP1–19 [26,27] and two MSP2 (*fcr3* and *3d7* alleles) polypeptides expressed in E. coli [28], and under the supervision of David Narum (Biomedical Primate Research Center, The Netherlands), using the AMA-1 recombinant protein, derived from clone 7G8 sequence, expressed in baculovirus, and hence properly conformed [27] (a gift of D. Narum and A. Thomas (Biomedical Primate Research Center, The Netherlands), ). Control peptides and recombinant proteins were chosen among pre-erythrocyte stage antigens, namely the peptides LSA1-R derived from liver stage antigen 1 [29], and LSA3-RE derived from liver stage antigen 3 [30], and the recombinant R32 LR derived from the circumsporozoite (CS) protein [31] spanning the NANP repeats.

### ELISAs

The total and isotype-specific ELISAs were performed as described previously [22,32,33] using secondary monoclonal antibodies originally selected as reacting faithfully with subclass allotypes of European, African, and Asian origins [22,32,33]. For IgG1 to IgG4 (IgG1–4), monoclonal mouse anti-human subclasses (clones NL16 = IgG1 [Boehringer], HP6002 = IgG2 [Sigma], Zg4 = IgG3, and RJ4 = IgG4 [both from Immunotech]) were used, at a final dilution of 1:2,000; 1:10,000; 1:10,000; and 1:1,000 respectively. For IgM (clone M11) determinations, the final dilution was 1:15,000. Each dilution of each monoclonal antibody had been previously determined as specifically reacting with the corresponding human isotype without showing significant cross-reaction with other isotypes [22,32,33].

All ELISA determinations were performed on coded serum samples, in a blinded manner—i.e., the investigators were not aware of the morbidity data of the corresponding patients.

The results were expressed first as ratios of OD values (or arbitrary units) as previously described [22,32,33] which were then used to estimate immunoglobulin concentrations. To calculate the OD ratios, each ELISA plate included sera from seven healthy French non-malaria-exposed blood donors, chosen as being representative of results obtained previously using sera from 200 French blood donors without exposure to malaria, i.e., yielding the same mean OD ± standard deviation (SD) value. The OD values corresponding to background responses from blood donors never exposed to malaria calculated in this manner were usually low, eg for MSP3: 0.035 (for IgG1); 0.050 (for IgG2); 0.027 (for IgG3); 0.031 (for IgG4); and 0.112 (for IgM). However, the precise cut-off value was calculated for each ELISA plate using the OD values recorded using the seven negative control sera included in each plate. The same positive and negative sera were systematically included in each 96 wells ELISA plate throughout the entire study. For the evaluation of immunoglobulin concentrations, the CLB reference serum with known amounts of each IgG subclass, IgM, and IgA (Central Laboratory of the Netherlands Red Cross blood transfusion service, Amsterdam, The Netherlands), and human reagents (either IgG myelomas or purified human IgM from Sigma) were used. The OD values obtained with our secondary monoclonal antibodies over a range of dilutions of the different reagents were used to determine the actual amounts of each immunoglobulin specific for each antigen present, and were converted to μg/ml equivalents.

### Analysis of Sequence Polymorphism in the *msp3* Gene

MSP3 PCR and sequencing focussed on the original DG 210 clone sequence corresponding to the C-terminal domain containing the three B cell epitope regions a, b, and c, as well as three T helper cell epitopes [23]. Forty-five P. falciparum isolates and strains were studied, obtained as follows: (i) ten from Kanbauk, Myanmar, Southeast Asia, where transmission is seasonal with between two and ten infective bites per year; (ii) 15 from Dielmo, Senegal, where transmission is holoendemic with 200 infective bites per year [16]; iii) ten from Ariquemes, State of Rondônia, Brazil, where transmission is perennial with 6.6 infective bites per month, and (iv) 13 laboratory strains—four from Tanzania, one from either Uganda, Liberia, Senegal, Thailand, Myanmar, Papua New Guinea, India, Brazil or Honduras and two from South East Asia. Parasite DNA from P. falciparum laboratory strains was extracted as described earlier [34]. DNA was extracted from blood samples using the QIAamp Blood Kit (Qiagen) in accordance with the manufacturer's instructions.

PCR and sequencing on parasite DNA was performed with primers DA151 (5′-G CCG GAA TTC CAT GAA AGG GCA AAA AAT GCT TA-3′) (nucleotides 562 to 585) (EcoRI cleavage site underlined) and DA152 (5′-G CCG GGA TCC ATT TTC CTT AGA TAT ATT TTC C-3′) (nucleotides 770 to 748) (BamHI cleavage site underlined). The program for PCR was a denaturation step at 94 °C for 1 min, an annealing step at 65 °C for 1 min followed by an extension step at 72 °C for 2 min and repeated for 35 cycles. PCR was performed using the GeneAmp PCR Kit (Perkin Elmer). The amplified products were purified by Spin-X (Costar) before sequencing on the ABI PRISM 373A DNA Sequencer using the ABI PRISM Dye Terminator Cycle Sequencing Ready Reaction Kit (Perkin Elmer). Nucleotide sequences were aligned by the CLUSTAL V program.

### Statistical Analysis

Data from 217 of the 247 Dielmo inhabitants were used, corresponding to those from whom serum samples had been collected. Thirty individuals, mostly adults, were excluded from the 247 because they spent less than half of their time in Dielmo during the follow-up, and were therefore deemed unsuitable for inclusion given that the degree of exposure plays a crucial role in the development and persistence of protective immunity. Of note, women who were pregnant for more than 10% of the study period were not included in the analysis and will be the subject of a separate report. A period of two years—one year before and one year after the serum sampling—was chosen for the initial clinical analysis, during which the number of malaria attacks was recorded. The villagers who were tested during a longer period of time, i.e., up to 6 y, corresponded to a subset of children and adults who stayed for more than 75% of their life in the village. The time spent in the village was determined on a continuous, active, and daily survey carried out by the medical team.

Information pertaining to the IgG1, IgG2, IgG3, IgG4, IgM, and IgA antibodies to MSP3, MSP1, MSP2 (FCR3), MSP2 (3D7), AMA1, and RESA; and IgG and IgM levels to LSA1-R, LSA3-RE, CS protein (R32LR recombinant protein), were thus available, as well as age, sex, number of days spent in the village, G6PD profile, and haemoglobin AS phenotype.

An inspection of the number of malaria attacks (identified over 2 y) plotted against the age of the individual affected, showed that they were not linearly distributed. A maximum-likelihood method was developed for adjustment of the data corresponding to malaria attacks. A nonlinear model was fitted to using a negative binomial loss function to calculate the breakpoint of threshold of age-dependent acquisition of immunity, for men and women separately in a manner similar to that described by Kocherlakota and Kocherlakota [35] (developed by K. Dietz, Department of Biomedical Biometry, University Eberhard-Karls of Tübingen, Tübingen, Germany). Even though a Poisson distribution also fitted the data, in the end a negative binomial was chosen, because it is frequently used to describe this type of data. Further assumptions were that every individual has a specific risk for a malaria attack. The distribution for the risk is, as conventionally used, a gamma distribution. The gamma distribution was standardized and had a mean of 1. The bivariate negative binomial distribution for both years had three parameters: a term for the variances (1/*k*) for the gamma distribution and the mean values for the two different years. The real risk of malaria in the different year is multiplicatory—and was given by a product term that consisted of an individual term and a year specific term, the latter being the same for all individuals. It was assumed that, for a particular individual, the number of attacks in the subsequent years was independently distributed according to a Poisson distribution with different means, which were proportional to the individual exposure risk.

The statistical models used for analysis of data were multivariate models. The potential relationship between malaria attacks (the variable to explain) and selected explanatory variables was tested using a log (1 + *x*) transformation of the number of attacks. The explanatory variables were selected on the basis of our existing knowledge of the potential involvement of these different parameters (with reference to our previous observations in different malaria-endemic areas). Age and age^2^, sex, haemoglobin phenotype and G6PD deficit as well as the individual immunoglobulin isotypes (i.e., IgG1, IgG2, IgG3, and IgG4) that were available and suspected of having a potential impact on the dependent variable (the number of malaria attacks), were tested in backward stepwise regression, eliminating, by hand, the variables which were not significant. The criterion for inclusion or elimination of the explanatory variables was that a predictor variable was included when its partial regression coefficient was significant at the 0.05 level and eliminated when its partial regression coefficient failed to be significant at the 0.1 level. The assumption that the regression equation accurately summarized the data was visually verified by examination of the residuals plotted against the fitted values. The significant terms were retained for further analysis and retested again to avoid exclusion due to interactions.

A negative binomial distribution is conventionally accepted for most disease data and has therefore been used to test the significant variables. Nominal and ordinal logistic regression (with no attack, one malaria attack, and two malaria attacks for the adults) and multivariate regression using a negative binomial distribution were used to analyse data with JMP software (SAS Institute, Cary, NC). For this analysis, IgG1 to IgG4 values were log-transformed or dichotomised as indicated in the ELISA section. A nonlinear model was also fitted to the significant variables of interest used to establish indications of long-term protection (between 1 and 6 y after the blood samples were tested) in children and adults of Dielmo. Chi-square and significance values were calculated looking at the difference between the full model and the model without the respective term. We used a level of significance of 0.05.

## Results

### Characterization of the Study Population

Of the 247 individuals constituting the whole village population of Dielmo, 217 were enrolled for the present study. The study cohort included 102 females (mean age ± SD 25.9 ± 21.1 y) and 115 males (21.9 ± 18.5 y). During the year preceding the blood sampling, the inhabitants of Dielmo involved in this study were present in the village during 78.2% ± 27.6% of the time (95% confidence interval [CI] 74.5%–82.0%). At the time of blood sampling, 112 inhabitants (mean age ± SD 30.5 ± 18.2 y) had a negative thick smear, whereas blood parasitaemia was detected in 55 individuals (21.8 ± 16.6 y) with less than 5,000 parasites/μl, and in 50 individuals (10.6 ± 10.7 y) who had ≥ 5,000 parasites/μl. In total, 130 villagers (30.8 ± 18.2 y) never presented with a malaria attack during the 2 y of follow-up and had a mean parasitaemia of 3,251 ± 10,841 parasites/μl (95% CI 1,553–4,949) at the time of sampling, whereas 87 (mean age ± SD 14.4 ± 18.3 y) had at least one malaria attack with a mean parasitaemia of 41,814 ± 191,964 parasites/μl (95% CI 0–88,638) at the time of the attack. The haemoglobin phenotype was AA in 191 individuals, AS in 23, and AC in 3; 46 villagers had a G6PD deficit.

### Identification of Clinical Cases and Evidence of Rapidly Acquired Protection in Young Children

**Figure 1:**
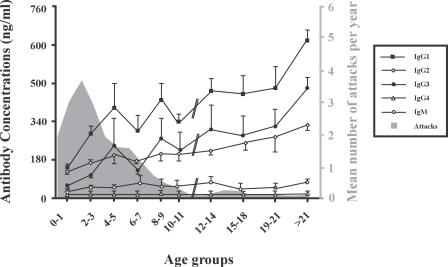
Means and Standard Deviations of Anti-MSP3-b IgG1, IgG2, IgG3, IgG4, and IgM Antibody Responses per Age Group Concentrations of antibodies are estimated as described in the Methods section. For each age group the mean number of malaria attacks recorded during the first year of follow-up is indicated by the shaded area. The numbers of individuals in each age group were: 5 (0–1 y), 14 (2–3 y), 17 (4–5 y), 14 (6–7 y), 12 (8–9 y), 13 (10–11 y), 11 (12–14 y), 18 (15–18 y), 13 19–21 y), 100 (>21 y), respectively.

The relevance of the analysis of immune responses critically depends on the reliability of clinical data. The daily clinical survey was satisfactorily carried out on 217 of the 247 individuals constituting the whole village population, who showed a classical age-dependent acquisition of clinical and parasitological immunity (Figure 1, shaded area). However, it was possible to distinguish, among the children, a subset of 14 individuals (20.9%) who did not present with an attack of malaria during the 2 y of follow-up (4/35 and 10/32 in the 0–5 y and 6–10 y age groups, respectively). Conversely, the study design also led to the identification of a small subset of 14 adolescents and adults (10.8%) occasionally susceptible to malaria attacks (3/28 and 11/101 in the 15–20 y and >21 y age groups, respectively), though the majority had acquired protection. However, in adults, symptoms were of short duration and resolved spontaneously, i.e., without requiring treatment in most cases [7,16,36].

The existence of such rapidly acquired protection in very young children and, conversely, the occurrence of brief malaria attacks in adults, have seldom been reported previously, possibly due to the use of less-stringent surveillance setups. The pattern of clinical incidence in Dielmo remains similar to that described in other high-endemicity African areas [37] with a far greater number of attacks in younger individuals, e.g., 3.45 clinical attacks per year in children aged 1–5 y versus 0.1 attack per year in adults. Nevertheless, the daily surveillance of highly exposed villagers made it possible to distinguish in every age group individuals with or without malaria attacks who can therefore be referred to below as “nonprotected” and “protected,” respectively, for the duration of the 2 y follow-up period. The monthly surveys of entomological inoculations, conducted during the clinical follow-up period, showed a very high level of transmission, with an estimate of 260 infective mosquito bites per person per year, i.e., 520 parasite inoculations over the 2 y survey, with little house to house variation, leading us to exclude the hypothesis that villagers without malaria attacks were not exposed.

This clinical situation was in most instances stable since only 13% of participants changed clinical status from the first to the second year of follow-up. The cases recorded are therefore well suited to the identification of immune correlates of protection and, furthermore, for the first time this analysis can be performed in an age-independent manner.

### Cytophilic Anti-MSP3 Antibodies Correlate with Clinical Immunity

The overall prevalence of IgG and IgM antibodies against the MSP3-b epitope in the 217 individuals studied was high (97.2% and 93.1%, respectively), whereas the prevalence of IgA was low (19.3%). MSP3-specific IgE antibodies could not be detected (unpublished data). Among the IgGs, the two cytophilic classes, IgG1 and IgG3, were the most abundant (Figure 1) and they both increased as a function of age, whereas the increase was modest for IgG2 and IgM antibodies, and low for IgG4 antibodies in all age groups.

Responses to other malaria antigens, including MSP1, MSP2-FC27, MSP2-3D7, RESA, and AMA-1, were also found to increase with age in this study (unpublished data) as has been observed in previous studies [38–42]. Therefore, due to this age-dependent increase, all antibodies measured showed an overall inverse relationship with the prevalence of clinical attacks (Figure 1, shaded area), a phenomenon reported in most previous studies in African settings and sometimes taken as indicating that protection is afforded by those antibodies [33,38,43–45]. The prevalence of responses to pre-erythrocytic antigens, such as CS, LSA1, and LSA3, also showed an age-dependent increase and confirmed the high degree of exposure to infected mosquitoes (see Figure S1). Antibodies against the pre-erythrocytic antigens were not included in the present statistical analysis.

We first sought by stepwise regression analysis if it was possible to select a subset of antibody responses that would tend to predict the number of malaria attacks when controlling for age. We then examined the predictive value in terms of clinical protection of a positive antibody response for each IgG isotype, to each of the five molecules studied, for both each year separately and combined. A highly consistent association was observed between protection and anti-MSP3 IgG3 antibodies for each of the years studied, as well as for the two years combined, and this indication persisted even when controlling for age (age-adjusted odds ratio [OR] and 95% CI = 7.19 [2.70–22.85], *p* < 0.001 for the 2 y of follow-up), whereas no similar association was found, when age was accounted for, for any of the other isotypes, nor for any of the various antibody responses to the other vaccine candidates (see Table S1). For example, for 2 y of survey, age-adjusted ORs and 95% CIs were, for anti-MSP1 IgG1, 0.36 (0.076–12.36), *p* = 0.134; for anti-MSP-1 IgG3, 1.38 (0.60–3.15), *p* = 0.437; for anti-AMA1 IgG3, 1.01 (0.41–2.40), *p* = 0.975; for anti-RESA IgG3, 1.18 (0.59–2.35), *p* = 0.637; for anti-MSP2 (FC27) IgG3, 0.74 (0.135–3.952), *p* = 0.727; and for anti-MSP2 (3D7), 0.81 (0.13–49), *p* = 0.823. The protected group had mean anti-MSP3 IgG3 values 3.75 times and 4.3 times higher than individuals having experienced either one or two or more attacks, respectively. The malaria attacks recorded over 2 y among the 177 individuals with anti-MSP3 IgG3 were 2.52-fold less frequent than among the 40 participants without detectable anti-MSP3 IgG3 responses.

**Figure 2:**
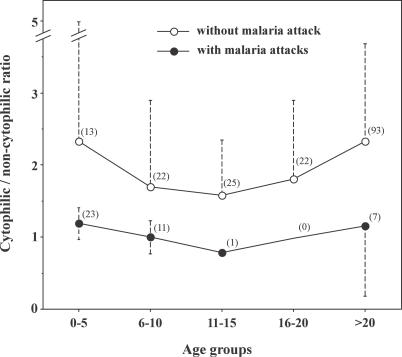
Ratios of Cytophilic to Noncytophilic Anti-MSP3 Responses Found Among Protected and Nonprotected Individuals of Dielmo The mean ratios of (IgG1 + IgG3) to (IgG2+ IgG4+ IgM) antibody responses (i.e., the cytophilic to noncytophilic [C:NC] ratios) were calculated for protected (open circles) and unprotected individuals (closed circles) within each age group. Error bars indicate the SD calculated for each age group. The number of individuals within each group is indicated in parentheses. A protected person is defined as an individual in whom no malaria attack was recorded during the first year of follow-up.

We have reported that it is the relative proportion of cytophilic antibodies to noncytophilic antibodies (the C:NC ratio) that is the most important surrogate marker of protection to date [32,46]. This is because anti-MSP3 antibodies act in a monocyte-dependent manner, requiring binding to Fc-γ receptors on monocytes [6,32]. The C:NC ratio takes into account the possible, and demonstrated, competition of noncytophilic antibodies directed towards the same epitope [15,32]. We found a consistently higher C:NC ratio, that is, a quantitative dominance of cytophilic classes, among protected as compared to nonprotected participants. This higher ratio was found in each age group (Figure 2), though it reached statistical significance only in the two youngest and the oldest age groups (0–5 y, *p* < 0.01; 6–10 y, *p* < 0.03; and >20, *p* < 0.007), probably due to group size. The overall C:NC ratio was significantly associated with protection for each year studied (OR [95% CI] = 2.79 [1.50–5.22], *p* = 0.001 for the year before sampling and 2.82 [1.51–5.30], *p* = 0.0012 for the year following the sampling), and for the two years combined (age-adjusted OR [95% CI] = 3.34 [1.72 – 6.67], *p* < 0.001). For the 2 y of survey, the other parameters were not significant, e.g., age-adjusted ORs (95% CIs) for the C:NC ratios were, for MSP1, 1.08 (0.57–2.11), *p* = 0.81; for AMA-1, 0.83 (0.43–1.61), *p* = 0.598; for RESA, 0.54 (0.13–2.04), *p* = 0.387; for MSP2 (FC27), 1.20 (0.43–3.38), *p* = 0.723; and for MSP2 (3D7), 1.35 (0.50–2.33), *p* = 0.551.

### IgG3 Anti-MSP3 Antibodies Are Strongly Associated with Clinical Protection against Malaria

Improved statistical analyses necessitated the use of larger group sizes. Since there was a clear age-dependent decrease in the number of attacks in children, whereas in adults the number of attacks was no longer age-dependent, we next determined the age threshold that informed the separation of individuals into two groups according to this criterion: for women this was 13.4 y and for men 17.9 y, over the 2 y observation period. These two groups were thus analysed in detail separately.

**Figure 3:**
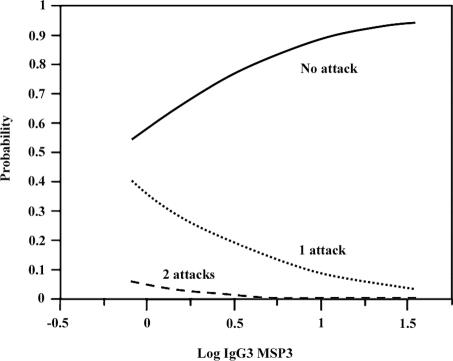
The Probability of No, One, or Two Malaria Attacks in the Adults of Dielmo as a Function of the Level of Anti-MSP3 IgG3 Responses The chance of no attack increased markedly and almost reached a maximum in parallel with the gradual increase in anti-MSP3b IgG3 responses. The probability that one or two malaria attacks occurred in adults decreased as the anti-MSP3b IgG3 responses increased.

Among the 121 individuals of the “adults” group, analyses of data by ordinal logistic regression showed that only MSP3 IgG3 and the C:NC ratio of MSP3 were associated with protection, i.e., negatively correlated with the number of malaria attacks (L-R Chi-square = 6.91; p = 0.0086 and L-R Chi-square = 10.84; *p* = 0.001 respectively). MSP1 IgG4 and AMA1 IgG3 were marginally significant if tested separately (*p* = 0.04 and 0.05 respectively), but were not significant if included in a model with MSP3 IgG3. No other variable showed any significance (unpublished data). In a nominal logistic regression, for MSP3 IgG3 the OR (95% CI) was 23.7 (3.15–238.36); and for the C:NC MSP3 ratio, it was 71.48 (3.65–1,339.68). The ORs for all other isotypes and other molecules did not reach significance. When the results were dichotomised, only MSP3 IgG3 (*p* = 0.022) and the C:NC ratio of MSP3 (*p* = 0.01) showed a significant relationship with protection. The surrogate value of anti-MSP3 IgG3 is graphically indicated in Figure 3, in which the risk of occurrence of one or two or more malaria attacks is shown to decrease in adults when the anti-MSP3 IgG3 levels increase.

Among the 96 individuals included in the “children” group, all variables were tested in a backward stepwise multivariate model with log-transformed isotype concentrations and then a negative binomial model fitted to the significant variables. The final model contained: (i) For IgG3: age (χ^2^ = 55.46, *p* < 0.001), MSP3 IgG3, (χ^2^ = 14.30; *p* < 0.001) and haemoglobin AS phenotype (χ^2^ = 5.56, p = 0.018), or (ii) For C:NC ratio: age (χ^2^ = 67.51; *p* < 0.001), MSP3 C:NC (χ^2^ = 12.21; *p* < 0.001), and haemoglobin AS phenotype (χ^2^ = 7.39; *p* = 0.0066). No other variable was found to be significant. For dichotomous antibody concentrations, MSP3 IgG3 (χ^2^ = 7.46, *p* = 0.0063) and MSP3 C:CN (χ^2^ = 4.036, *p* = 0.045) were also significant. The OR (95% CI), when tested in a nominal logistic regression model, was for age, 51.15 (6.71–515.37), *p* < 0.001; and for MSP3 IgG3, 15.49 (1.57–184.65), *p* = 0.0219 (for age 96.93 [12.16–1093.25], *p* < 0.001 and for MSP3 C:NC 64.19 [2.94–2,204.40], *p* = 0.0118). Results from an ordinal regression model with no attack, one, or more than one attack were similar to the results from the previous model except that haemoglobin AS phenotype was no longer significant.

### The Clinical Significance of IgG3 Anti-MSP3 Antibodies Extends to Several Years

In view of the above results, which show a strong association of the surrogate marker with clinical protection, and since individuals in the village were followed up clinically in the ensuing years, we next addressed the question of the long-term clinical significance of a single antibody determination.

Individuals resident in Dielmo for more than 75% of the time in the ensuing 6 y after the initial blood sampling were selected for this analysis. For this reason, the number of individuals included in the 6 y analysis is lower than in the 2 y analysis.

In the adult group, the association of the IgG3 anti-MSP3 responses with protection was significant for the year following sampling (OR 14.1, *p* = 0.019) but no longer significant, in the ensuing years (as shown in Table S2). This indication was not found for anti-MSP1 IgG3, with OR (95% CI) of 7.27 (0.85–145.35), *p* = 0.111; for anti-AMA1 IgG3, 2.92 (0.19–303.21), *p* = 0.527; for anti-RESA IgG3, 2.67 (0.68–10.62), *p* = 0.157; nor for anti-MSP2 (FC27) IgG3, 0.78 (0.04–6.16), *p* = 0.832.

In contrast, in the children group, a significant association of IgG3 anti-MSP-3 antibodies with clinical protection was observed in the following 12, 24, and 36 mo of follow-up (respective ORs 56.1, *p* = 0.0063; 34.8, *p* = 0.01, and 31.7, *p* = 0.009). Only by year 4 and onwards did the clinical value decrease, though it remained significant (OR, 15.49, *p* = 0.037; 18.2, *p* = 0.031, and 21, *p* = 0.036 on years 4, 5, and 6, respectively, as shown in Table S2). Of note, a similar analysis did not lead to the same observations for antibodies specific to other antigens (for example, after 1 y of follow-up, it was found in the children group, for anti-MSP1 IgG3, 1.30 [0.11–29.67], *p* = 0.84; for anti-AMA1 IgG3, 0.93 [0.18–4.9], *p* = 0.931; for anti-RESA IgG3, 1.89 [0.52–7.75], *p* = 0.345; for anti-MSP2 (FC27) IgG3, 0.20 [0.015–1.918], *p* = 0.183; and for anti-MSP2 (3D7), 0.71 [0.05–9.14], *p* = 0.785).

The above results were confirmed using a nonlinear model fitted to the variables demonstrated to be significant in the previous analysis, i.e., age, haemoglobin AS phenotype, and anti-MSP-3 IgG3. The maximum difference between the mean number of malaria attacks identified in children with anti-MSP-3 IgG3, and children without such a response (4.3-fold), was detected 3 y after sampling (χ^2^ = 11.76; *p* = 0.0006) and remained marked by year 4 of the study (χ^2^ = 9.76; *p* = 0.0017). The differential number of attacks was still significant by year 6 of the follow-up (χ^2^ = 4.64; *p* = 0.031) when the model was compared with and without the antibody response. Hence, the two methods of analysis provided convergent conclusions indicative of the long-term clinical significance of a single IgG3 anti-MSP3b determination, in terms of the resistance of children to malaria attacks.

**Figure 4:**
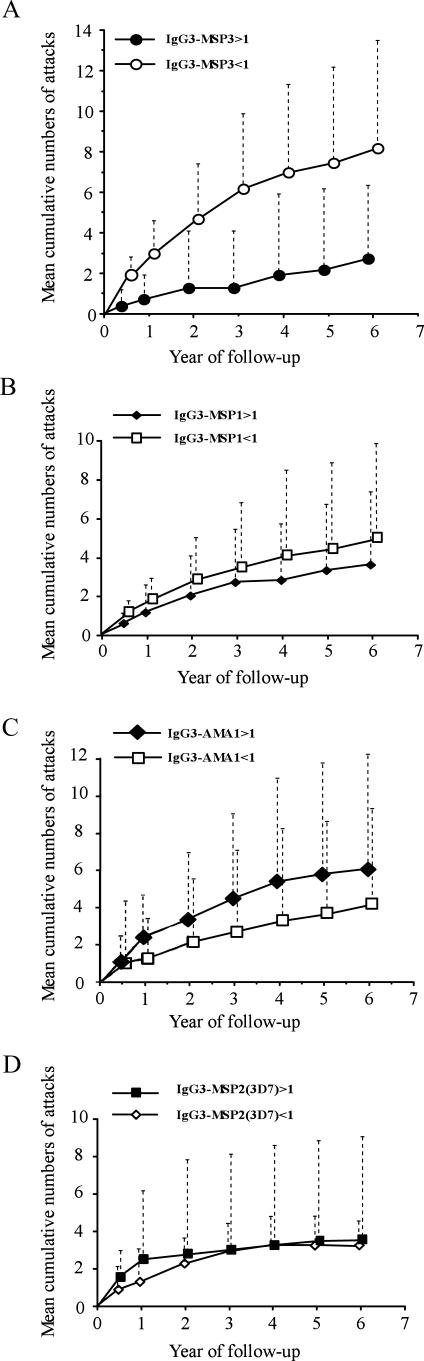
Mean Cumulative Number of Malaria Attacks Identified over Six Years of Follow-up in Dielmo, in Relation to IgG3 Antibodies to Each Antigen For each antigen, specific IgG3 responses were dichotomized (antibody ratio less than or greater than 1) and in each group the occurrence of malaria attacks was calculated. To avoid the confounding effect of age, a subgroup of 49 children was selected for this analysis so that the mean age was similar (5.5 ± 2.6 y) among children with or without antibodies to each antigen. The mean cumulative numbers of malaria attacks are indicated for anti-MSP3b (A), anti-MSP1 (B), anti-AMA1 (C), and anti-MSP2-3D7 (D) IgG3 responses with ratio values less than 1 (open circles) or greater than 1 (closed circles). Error bars indicate SD.

Results are graphically illustrated in Figure 4, which shows the cumulative number of malaria attacks over 6 y of follow-up in Dielmo, in relation to IgG3 antibodies to each antigen. When the analysis was performed comparing the same individuals with either “low” or “high” antibody levels (IgG3 levels below or above the median), similar conclusions were reached (see Figure S2).

### The Target Epitope of Antibodies Associated with Protection Is Fully Conserved in Various Parasite Isolates

Since MSP3 appeared at this point to be a strong surrogate marker of clinical protection, we deemed it important to investigate whether this antigen exhibits the polymorphisms reported for many malarial vaccine candidates [47–50]. The sequence of the C terminus part of the gene, encoding three B cell and four Th cell epitopes [23] was analyzed in 45 parasite strains and isolates and did not reveal any polymorphism, either at the amino acid level or at the nucleotide level (unpublished data). The importance of this result is supported by the parallel study of the nonrepetitive 3′ region of the *CS* gene (nucleotides 1,015 to 1,254), which revealed 16 nucleotide substitutions in the same 45 strains (ten within the Th2R and six within the Th3R regions), all resulting in amino acid substitutions, in agreement with previous reports [51].

## Discussion

A detailed analysis of the malarial clinical status, based on daily medical records actively collected from each inhabitant of Dielmo, Senegal, demonstrated a highly significant relationship between clinical protection acquired through natural exposure and the production of cytophilic anti-MSP-3 antibodies, notably IgG3. The stronger relationship of this marker with protection than that conferred by sickle cell trait (i.e., S-type haemoglobin) indicates that its contribution to the control of parasites is highly significant and therefore demonstrates its promise as a vaccine candidate. Above all, the clinical value of this surrogate marker, i.e., its association with protection, persisted out to six years in individuals who are exposed to approximately 260 inoculations per year of parasites by *Anopheles* mosquitoes [16].

Except for one study in which two antigens were investigated in parallel [52], most immunoepidemiological studies have addressed the surrogate value of antibodies only to single vaccine candidates, each studied in distinct setups and frequently with distinct criteria, thereby precluding comparative analysis of the clinical significance of immune responses to each antigen. In the comparative analysis of five antigens that we performed, the involvement of natural antibody responses to the four other antigens studied simultaneously did not reach significance. In other words, out of 30 different combinations of isotype-specific responses determined for each of the inhabitants tested, only one was strongly associated with naturally acquired protection. This observation is of particular value, given the problem of colinearity of immune responses, which could either hide this relationship or bias the analysis towards the role of other antibody responses. The other immune responses were not associated with protection. This obviously does not mean that they are not important, but that the conditions of our study and the power of our analysis within the limitations of group size cause the results to fail to reach significance for these other parameters.

In such epidemiological investigations, age is a confounding factor, since protection builds up over time with exposure, and hence with age [53,54]. This variable could be fully controlled in our setup for the first time as both protected and nonprotected individuals were identified in each age group by our study design. That some children can rapidly acquire a potent and long-lasting level of protection against the disease is a novel finding that has important consequences: since protection induced by natural exposure can develop early in life, it raises the realistic hope that it should be feasible to induce protection at an early time point by vaccination. The mechanisms leading to sustained protection over the years in some children remain hypothetical. The strong association with protection of IgG3 responses in younger individuals is reminiscent of similar findings made previously in Dielmo [55]. This confirmation suggests that some children can achieve an early isotype switch towards cytophilic responses, and thereby can remain free of malaria attacks for extended periods of time.

Following the report that the isotype distribution of antimalarial antibodies was a critical parameter of malaria immunity [46], various studies confirmed the increase of IgG3 with age, and hence with protection, for many malarial antigens including SIRBC extract, MSP1, MSP2, AMA1, RESA, GLURP, p126-SERP-SERA, EBA 175, TRAP, and Exp1 [28,38,39,45,52,55–62]. In these studies where, with few exceptions [63], the children were collectively considered nonprotected and the adults protected, the increase in antibody levels with increasing age and increased protection led to the report of an association between protection to malaria and antibody to antigens including MSP1–19 [64], MSP1 Block2 [65], SERP [57], and GLURP [56]. The absence of any correlation between anti-MSP1–19 antibodies and protection demonstrated herein is in agreement with some [44] but not all previous reports [66,67]. One study suggested that the risk of malaria is reduced only when IgG antibodies to MSP1–19 are present at very high titters [42]; however, more recent work concluded that only antibodies inhibiting the processing of MSP1, but not those directed to MSP1–19, were associated with protection [42,68]. These processing-inhibitory antibodies were not investigated in our study. Responses to the highly polymorphic MSP1-Block2 region have also been reported to be associated with protection; however, when corrected for age this effect was shown to be mainly related to one of the 16 polymorphic constructs inducing low titters of antibodies in a small subset of individuals. Moreover, responses waned over time and became negligible at the end of the first year, in contrast to antibodies to MSP3. Recently it was reported that polymorphisms in the central and N-terminal region of MSP3 appeared as a result of immune selection. In that study, antibodies to both the polymorphic region and the C terminus (which includes the peptide MSP3b) were found to be significantly associated with naturally acquired protection [69]. Although only total IgG was evaluated (and therefore the critical role of IgG3 could not be identified) and the clinical data were collected over a shorter period of 5 mo, this result is in agreement with our current findings and supports the clinical value of anti-MSP3 Abs. Results reported elsewhere [69,58] also show that under conditions of follow-up less stringent than in Dielmo, similar conclusions were reached about the association of anti-MSP3 antibodies with protection and in other settings such as The Gambia and Myanmar [69,58], i.e., with distinct human and parasite genetic backgrounds, and much lower transmission conditions.

The long-term clinical effect of a single anti-MSP3 antibody determination over a number of years in participants that are receiving malaria inoculations by mosquitoes every two days is impressive, particularly as it remained significant over six consecutive years. This finding has not been reported for any other antigen, to our knowledge. The conceptual importance and implications for vaccine development merit further investigation in other settings. The longer-term association in children than in adults is likely related to two factors: (i) children can be divided into two groups, a small subset acquiring protection early in life who remain protected in the ensuing years (this is associated with our surrogate marker) and the majority who do not, and (ii) most adults are continuously protected against malaria, but some may briefly lose this protection and reacquire it after a short, self-resolving attack. A long-term effect cannot be expected in these occasional situations of brief loss of existing protection.

Malaria vaccine discovery, which has relied for several decades on preclinical in vitro and in vivo animal models, is now increasingly reliant on clinical trials in humans. For two major reasons this is driving vaccine development towards an ever more unmanageable situation. One reason is that since none of the preclinical models is recognised as being of undisputable predictive value, each candidate must presently be taken into lengthy, time-consuming and costly clinical trials. From an initial number of 12 vaccine candidates, this translates, with various antigen presentation systems, into 58 planned clinical trials (http://www.who.int/vaccine_research/documents/en/malaria_table.pdf) and a concomitant increase in development costs. The second reason is that the elucidation of the malaria genome, although welcome, nonetheless generates knowledge about ∼5,300 proteins—500-fold more molecules than was previously known. These potential antigens must now be assessed for their suitability for inclusion in a malaria vaccine, a task that is not achievable through individual vaccine efficacy trials with each one.

The immunoepidemiological screening approach that we have taken has the advantage of relying on immune responses that occur in the ultimate target of a vaccine, namely human beings. Hence, we postulate that this approach provides results that are more relevant and ultimately more reliable than those derived from any experimental model. It also has the advantage of being rapid and applicable to any number of antigens, with the sole drawback of not identifying antigens or mechanisms that do not exist in nature. Our study indicates that this method can point to major differences among vaccine candidates when these are studied under comparative conditions, and therefore provides one clue to the complex equation malaria vaccine discovery is now facing.

The present study merely shows an association, i.e., value as a surrogate marker, of anti-MSP3 antibodies with acquired protection to malaria in exposed populations. However, this immunoepidemiological result also agrees with results from a series of experimental studies, conducted in vitro and in vivo, that showed an antiparasitic effect of the same antibodies. Human anti-MSP3b polyclonal [22,23,70,71] or monoclonal antibodies [72,73], or animal anti-MSP3b antibodies [22,70], were consistently found to exert a strong antiparasitic effect in vitro with blood monocytes. It is noteworthy that the IgG3 version of a human recombinant antibody against MSP3b was actually more effective in vitro than was the IgG1 version, and that in the present independent immunoepidemiological approach a greater association of the same isotype to the same epitope is found [73]. Upon passive transfer in vivo in a humanised mouse model, anti-MSP3b antibodies fully cleared P. falciparum parasitaemia, using either naturally occurring or vaccine-induced human antibodies [23,70,71]. Finally, vaccination of primates with MSP3 induced the strongest in vivo protection observed in this model of human malaria against a P. falciparum challenge [74]. Although anti-MSP3 IgG3 may be only a marker, associated with but not directly responsible for protection, there is a remarkable convergence between experimental data acquired under in vitro conditions or in animal models, with observations of immune responses in exposed human populations.

The full sequence conservation of the B cell epitopes from the C-terminal region confirms previous results [24,25] and contrasts with the polymorphism found in other regions of MSP3 and in other antigens, such as CS, TRAP, MSP1, and MSP2. We have previously discussed why an indirect mechanism such as ADCI is consistent with the phenomenon of chronicity and why it does not lead, in contrast to direct mechanisms of action of antibodies, to antigen polymorphisms [75]. In this respect it seems debatable to employ the most polymorphic part of MSP3 for vaccine studies, as was proposed recently [76], particularly since the conserved C terminus has now demonstrated its ability to elicit in humans antibodies that kill P. falciparum [71]. The C-terminal part of MSP3 has structural homology with MSP6, and has sequence identities (ILGWEFGGGA/VP) within the MSP3b peptide region resulting in fully cross-reactive epitopes between the two molecules [77]. This observation and the recent finding that these two molecules belong to a multigene family with structural and immunological homologies (S. Singh et al., unpublished data), reinforce the immunological importance of the MSP3b epitope and make it understandable that knocking out one of the family members has few functional consequences [78]. Conservation of the sequence across isolates and duplication of the sequence in other genes are of evident importance whether MSP3 constitutes only a surrogate marker as shown here and in previous studies [22,23,58,69–71,79] or whether, as suggested elsewhere, it also has vaccine potential against P. falciparum [70,71,74,80]. We therefore hope that the present results will trigger the implementation of more immunoepidemiological studies to further document this important finding.

## Supporting Information

Figure S1 Antibody Responses to Pre-erythrocytic Antigens among Dielmo Inhabitants Prevalence (% positives) to various pre-erythrocytic antigens among 217 inhabitants from Dielmo (Senegal) was measured by ELISA against LSA1-, LSA3-, and SALSA-derived peptides, two recombinant proteins (R32 LR and R32Tet), and a peptide (NANP4) derived from the CS protein.
(6.9 MB EPS)

Figure S2 Cumulative Number of Malaria Attacks over 6 Years of Follow-up in Dielmo in Individuals with Either High or Low Antibody Responses to Four Leading Malaria Vaccine Candidates For each antigen, the median value of antibody responses was determined. Individuals with antibody responses above the median (>median) were considered high responders (closed circles). Individuals with antibody responses below the median (<median) were considered low responders (open circles). Following a single determination of antibodies to each antigen in blood samples, the mean number of malaria attacks recorded in Dielmo over 6 y was calculated for each year of the study and for each group of children (either high or low responders to each antigen). The two subgroups had the same mean age, so that there was no age imbalance between children with and without malaria attacks. The mean cumulative numbers of malaria attacks are illustrated for anti-MSP3b (A), anti-MSP1 (B), anti-AMA1 (C) and anti-MSP2-3D7 (D) IgG3 responses. Error bars indicate SD.
(1.1 MB EPS)

Table S1 Results of Univariate Analysis of Antibody Responses in Relation to Malaria Attacks ORs and 95% CIs were calculated to evaluate the relationship between two binary variables (i.e., presence or absence of a positive antibody response and occurrence or absence of malaria attack during the 2 y of follow-up). The results are given as an indication of effect size with ORs greater than 1 indicating that the no malaria attack condition was more likely to happen in the group with antibody responses specific for the antigen tested. C:NC indicates the cytophilic to noncytophilic ratios (i.e., the ratios of [IgG1 + IgG3] to[IgG2 + IgG4 + IgM] antibody responses); NA (not available) indicates situations where ORs could not be calculated.
(1.2 MB EPS)

Table S2 Association between IgG3 anti-MSP3 Responses and the Cumulative Number of Malaria Attacks over Years A subgroup of Dielmo inhabitants present during 6 y of survey after blood sampling was identified. Presence or absence of anti-MSP3 IgG3 responses was tested with regard to the cumulative number of malaria attacks identified each year. The indications in favour of a potential association between anti-MSP3 IgG3 responses and resistance to malaria attacks recorded during 6 mo to 6 y following blood sampling are given as age-adjusted ORs and 95% CIs determined for children and adults separately.
(482 KB EPS)

### Accession Numbers

The primer positions in the MSP3seqence are based on GenBank (http://www.ncbi.nlm.nih.gov/) accession number AF024624.

## Abbreviations

CI: confidence interval
CS: circumsporozoite
MSP: merozoite surface protein
OR: odds ratio
SD: standard deviation

## References

[pmed-0040320-b001] Graves P, Gelband H (2003). Vaccines for preventing malaria. Cochrane Database Syst Rev.

[pmed-0040320-b002] Webster D, Hill AV (2003). Progress with new malaria vaccines. Bull World Health Organ.

[pmed-0040320-b003] Druilhe P, Hagan P, Rook GA (2002). The importance of models of infection in the study of disease resistance. Trends Microbiol.

[pmed-0040320-b004] Taylor-Robinson AW (2002). A model of development of acquired immunity to malaria in humans living under endemic conditions. Med Hypotheses.

[pmed-0040320-b005] Cohen S, Mc Gregor A, Carrington S (1961). Gamma globulin and acquired immunity to human malaria. Nature.

[pmed-0040320-b006] Bouharoun-Tayoun H, Attanah P, Sabchareon A, Chongsuphajaisiddhi T, Druilhe P (1990). Antibodies that protect humans against Plasmodium falciparum blood stages do not on their own inhibit parasite growth and invasion in vitro, but act in cooperation with monocytes. J Exp Med.

[pmed-0040320-b007] Rogier C, Commenges D, Trape J (1996). Evidence for an age-dependent pyrogenic threshold of Plasmodium falciparum parasitemia in highly endemic populations. Amer J Trop Med Hyg.

[pmed-0040320-b008] Gatton ML, Cheng Q (2002). Evaluation of the pyrogenic threshold for Plasmodium falciparum malaria in naive individuals. Am J Trop Med Hyg.

[pmed-0040320-b009] Boutlis CS, Weinberg JB, Baker J, Bockarie MJ, Mgone CS (2004). Nitric oxide production and nitric oxide synthase activity in malaria-exposed Papua New Guinean children and adults show longitudinal stability and no association with parasitemia. Infect Immun.

[pmed-0040320-b010] Egan AF, Burghaus P, Druilhe P, Holder AA, Riley EM (1999). Human antibodies to the 19 kDa C-terminal fragment of Plasmodium falciparum merozoite surface protein 1 inhibit parasite growth in vitro. Parasite Immunol.

[pmed-0040320-b011] Okoyeh JN, Pillai CR, Chitnis CE (1999). Plasmodium falciparum field isolates commonly use erythrocyte invasion pathways that are independent of sialic acid residues of glycophorin A. Infect Immun.

[pmed-0040320-b012] Triglia T, Healer J, Caruana SR, Hodder AN, Anders RF (2000). Apical membrane antigen 1 plays a central role in erythrocyte invasion by *Plasmodium* species. Mol Microbiol.

[pmed-0040320-b013] Casey JL, Coley AM, Anders RF, Murphy VJ, Humberstone KS (2004). Antibodies to malaria peptide mimics inhibit Plasmodium falciparum invasion of erythrocytes. Infect Immun.

[pmed-0040320-b014] Contreras CE, Santiago JI, Jensen JB, Udeinya IJ, Bayoumi R (1988). RESA-IFA assay in Plasmodium falciparum malaria, observations on relationship between serum antibody titers, immunity, and antigenic diversity. J Parasitol.

[pmed-0040320-b015] Bouharoun-Tayoun H, Oeuvray C, Lunel F, Druilhe P (1995). Mechanisms underlying the monocyte-mediated antibody-dependent killing of *Plasmodium falciparum* asexual blood stages. J Exp Med.

[pmed-0040320-b016] Trape JF, Rogier C, Konate L, Diagne N, Bouganali H (1994). The Dielmo project. A longitudinal study of natural malaria infection in a community living in a holoendemic area of Senegal. Am J Trop Med Hyg.

[pmed-0040320-b017] Rogier C (2000). Natural history of Plasmodium falciparum malaria and determining factors of the acquisition of antimalaria immunity in two endemic areas, Dielmo and Ndiop (Senegal). Bull Mem Acad R Med Belg.

[pmed-0040320-b018] McGuinness D, Koram K, Bennett S, Wagner G, Nkrumah F (1998). Clinical case definitions for malaria: clinical malaria associated with very low parasite densities in African infants. Trans R Soc Trop Med Hyg.

[pmed-0040320-b019] Bouvier P, Rougemont A, Breslow N, Doumbo O, Delley V (1997). Seasonality and malaria in a west African village: does high parasite density predict fever incidence?. Am J Epidemiol.

[pmed-0040320-b020] Smith T, Schellenberg JA, Hayes R (1994). Attributable fraction estimates and case definitions for malaria in endemic areas. Stat Med.

[pmed-0040320-b021] Trape J, Peelman P, Morault-Peelman B (1985). Criteria for diagnosing clinical malaria among a semi-immune population exposed to intense and perennial transmission. Trans Roy Soc Trop Med Hyg.

[pmed-0040320-b022] Oeuvray C, Bouharoun-Tayoun H, Gras-Masse H, Bottius E, Kaidoh T (1994). Merozoite surface protein-3: a malaria protein inducing antibodies that promote *Plasmodium falciparum* killing by cooperation with blood monocytes. Blood.

[pmed-0040320-b023] Singh S, Soe S, Mejia JP, Roussilhon C, Theisen M (2004). Identification of a conserved region of Plasmodium falciparum MSP3 targeted by biologically active antibodies to improve vaccine design. J Infect Dis.

[pmed-0040320-b024] McColl D, Anders R (1997). Conservation of structural motifs and antigenic diversity in the Plasmodium falciparum merozoite surface protein-3 (MSP-3). Mol Biochem Parasitol.

[pmed-0040320-b025] Huber W, Felger I, Matile H, Lipps H, Steiger S (1997). Limited sequence polymorphism in the Plasmodium falciparum merozoite surface protein 3. Mol Biochem Parasitol.

[pmed-0040320-b026] Chappel J-A, Egan AF, Riley EM, Druilhe P, Holder AA (1994). Naturally acquired human antibodies which recognise the first epidermal growth factor-like module in the Plasmodium falciparum merozoite surface protein-1 do not inhibit parasite growth in vitro. Infec Immun.

[pmed-0040320-b027] Thomas A, Trape J, Rogier C, Goncalves A, Rosario V (1994). High prevalence of natural antibodies against Plasmodium falciparum 83-kilodalton apical membrane antigen (PF83/AMA-1) as detected by capture-enzyme-linked immunosorbent assay using full-length baculovirus recombinant PF83/AMA-1. Amer J Trop Med Hyg.

[pmed-0040320-b028] Rzepczyk CM, Hale K, Woodroffe N, Bobogare A, Csurhes P (1997). Humoral immune responses of Solomon Islanders to the merozoite surface antigen 2 of Plasmodium falciparum show pronounced skewing towards antibodies of the immunoglobulin G3 Subclass. Infec Immun.

[pmed-0040320-b029] Fidock DA, Gras-Masse H, Lepers JP, Brahimi K, Benmohamed L (1994). Plasmodium falciparum liver stage antigen-1 is well conserved and contains potent B and T cell determinants. J Immunol.

[pmed-0040320-b030] Daubersies P, Thomas AW, Millet P, Brahimi K, Langermans JA (2000). Protection against Plasmodium falciparum malaria in chimpanzees by immunization with the conserved pre-erythrocytic liver-stage antigen 3. Nat Med.

[pmed-0040320-b031] Brown AE, Webster HK, Gordon DM, Permpanich B, Gross M (1992). Characterization of naturally acquired antibodies to the non-repetitive flanking regions of the circumsporozoite protein of Plasmodium falciparum. Am J Trop Med Hyg.

[pmed-0040320-b032] Bouharoun-Tayoun H, Druilhe P (1992). Plasmodium falciparum malaria: evidence for an isotype imbalance which may be responsible for delayed acquisition of protective immunity. Infect Immun.

[pmed-0040320-b033] Theisen M, Soe S, Oeuvray C, Thomas AW, Vuust J (1998). The glutamate-rich protein (Glurp) of Plasmodium falciparum is a target for antibody-dependent monocyte-mediated inhibition of parasite growth in vitro. Infec Immun.

[pmed-0040320-b034] Petersen E, Borre M, Vuust J (1991). Allele specific analysis of Plasmodium falciparum genes by the polymerase chain reaction. Mol Immunol.

[pmed-0040320-b035] Kocherlakota S, Kocherlakota K (1992). Bivariate discrete distributions.

[pmed-0040320-b036] Rogier C, Ly AB, Tall A, Cisse B, Trape JF (1999). Plasmodium falciparum clinical malaria in Dielmo, a holoendemic area in Senegal: no influence of acquired immunity on initial symptomatology and severity of malaria attacks. Am J Trop Med Hyg.

[pmed-0040320-b037] Marsh K, Snow RW (1999). Malaria transmission and morbidity. Parassitologia.

[pmed-0040320-b038] Taylor RR, Allen SJ, Greenwood BM, Riley EM (1998). IgG3 antibodies to *Plasmodium falciparum* merozoite surface protein 2 (MSP2): increasing prevalence with age and association with clinical immunity to malaria. Am J Trop Med Hyg.

[pmed-0040320-b039] Polley SD, Mwangi T, Kocken CH, Thomas AW, Dutta S (2004). Human antibodies to recombinant protein constructs of Plasmodium falciparum apical membrane antigen 1 (AMA1) and their associations with protection from malaria. Vaccine.

[pmed-0040320-b040] al-Yaman F, Genton B, Anders R, Taraika J, Ginny M (1995). Assessment of the role of the humoral response to Plasmodium falciparum MSP2 compared to RESA and SPf66 in protecting Papua New Guinean children from clinical malaria. Parasite Immunol.

[pmed-0040320-b041] Chizzolini C, Dupont A, Akue JP, Kaufmann MH, Verdini AS (1988). Natural antibodies against three distinct and defined antigens of Plasmodium falciparum in residents of a mesoendemic area in Gabon. Am J Trop Med Hyg.

[pmed-0040320-b042] Perraut R, Marrama L, Diouf B, Sokhna C, Tall A (2005). Antibodies to the conserved C-terminal domain of the Plasmodium falciparum merozoite surface protein 1 and to the merozoite extract and their relationship with in vitro inhibitory antibodies and protection against clinical malaria in a Senegalese village. J Infect Dis.

[pmed-0040320-b043] Marsh K, Otoo L, Hayes RJ, Carson DC, Greenwood BM (1989). Antibodies to blood stage antigens of Plasmodium falciparum in rural Gambians and their relation to protection against infection. Trans R Soc Trop Med Hyg.

[pmed-0040320-b044] Dodoo D, Theisen M, Kurtzhals JA, Akanmori BD, Koram KA (2000). Naturally acquired antibodies to the glutamate-rich protein are associated with protection against Plasmodium falciparum malaria. J Infect Dis.

[pmed-0040320-b045] Topolska AE, Richie TL, Nhan DH, Coppel RL (2004). Associations between responses to the rhoptry-associated membrane antigen of Plasmodium falciparum and immunity to malaria infection. Infect Immun.

[pmed-0040320-b046] Bouharoun-Tayoun H, Druilhe P (1992). Antibodies in *falciparum* malaria: what matters most, quantity or quality?. Mem Inst Oswaldo Cruz, Rio de Janeiro.

[pmed-0040320-b047] Healer J, Murphy V, Hodder AN, Masciantonio R, Gemmill AW (2004). Allelic polymorphisms in apical membrane antigen-1 are responsible for evasion of antibody-mediated inhibition in Plasmodium falciparum. Mol Microbiol.

[pmed-0040320-b048] Sakihama N, Matsuo T, Mitamura T, Horii T, Kimura M (2004). Relative frequencies of polymorphisms of variation in block 2 repeats and 5′ recombinant types of *Plasmodium falciparum msp1* alleles. Parasitol Int.

[pmed-0040320-b049] Aubouy A, Migot-Nabias F, Deloron P (2003). Polymorphism in two merozoite surface proteins of Plasmodium falciparum isolates from Gabon. Malar J.

[pmed-0040320-b050] Escalante AA, Grebert HM, Chaiyaroj SC, Magris M, Biswas S (2001). Polymorphism in the gene encoding the apical membrane antigen-1 (AMA-1) of Plasmodium falciparum. X. Asembo Bay Cohort Project. Mol Biochem Parasitol.

[pmed-0040320-b051] de Stricker K, Vuust J, Jepsen S, Oeuvray C, Theisen M (2000). Conservation and heterogeneity of the glutamate-rich protein (GLURP) among field isolates and laboratory lines of Plasmodium falciparum. Mol Biochem Parasitol.

[pmed-0040320-b052] Metzger WG, Okenu DM, Cavanagh DR, Robinson JV, Bojang KA (2003). Serum IgG3 to the Plasmodium falciparum merozoite surface protein 2 is strongly associated with a reduced prospective risk of malaria. Parasite Immunol.

[pmed-0040320-b053] Sergent ED, Parrot L (1935). L'immunité, la prémunition et la résistance innée. Archives de l'institut Pasteur d'Algérie.

[pmed-0040320-b054] Rogier C, Trape JF (1993). Malaria attacks in children exposed to high transmission: who is protected?. Trans R Soc Trop Med Hyg.

[pmed-0040320-b055] Aribot G, Rogier C, Sarthou JL, Trape JF, Balde AT (1996). Pattern of immunoglobulin isotype response to Plasmodium falciparum blood-stage antigens in individuals living in a holoendemic area of Senegal (Dielmo, West Africa). Am J Trop Med Hyg.

[pmed-0040320-b056] Oeuvray C, Theisen M, Rogier C, Trape JF, Jepsen S (2000). Cytophilic immunoglobulin responses to Plasmodium falciparum glutamate-rich protein are correlated with protection against clinical malaria in Dielmo, Senegal. Infect Immun.

[pmed-0040320-b057] Okech BA, Nalunkuma A, Okello D, Pang XL, Suzue K (2001). Natural human immunoglobulin G subclass responses to Plasmodium falciparum serine repeat antigen in Uganda. Am J Trop Med Hyg.

[pmed-0040320-b058] Soe S, Theisen M, Roussilhon C, Aye KS, Druilhe P (2004). Association between protection against clinical malaria and antibodies to merozoite surface antigens in an area of hyperendemicity in Myanmar: complementarity between responses to merozoite surface protein 3 and the 220-kilodalton glutamate-rich protein. Infect Immun.

[pmed-0040320-b059] Okenu DM, Riley EM, Bickle QD, Agomo PU, Barbosa A (2000). Analysis of human antibodies to erythrocyte binding antigen 175 of Plasmodium falciparum. Infect Immun.

[pmed-0040320-b060] John CC, Zickafoose JS, Sumba PO, King CL, Kazura JW (2003). Antibodies to the Plasmodium falciparum antigens circumsporozoite protein, thrombospondin-related adhesive protein, and liver-stage antigen 1 vary by ages of subjects and by season in a highland area of Kenya. Infect Immun.

[pmed-0040320-b061] Meraldi V, Nebie I, Tiono AB, Diallo D, Sanogo E (2004). Natural antibody response to Plasmodium falciparum Exp-1, MSP-3 and GLURP long synthetic peptides and association with protection. Parasite Immunol.

[pmed-0040320-b062] Beck HP, Felger I, Genton B, Alexander N, al-Yaman F (1995). Humoral and cell-mediated immunity to the Plasmodium falciparum ring-infected erythrocyte surface antigen in an adult population exposed to highly endemic malaria. Infect Immun.

[pmed-0040320-b063] Conway DJ, Cavanagh DR, Tanabe K, Roper C, Mikes ZS (2000). A principal target of human immunity to malaria identified by molecular population genetic and immunological analyses. Nat Med.

[pmed-0040320-b064] de Koning-Ward TF, O'Donnell RA, Drew DR, Thomson R, Speed TP (2003). A new rodent model to assess blood stage immunity to the Plasmodium falciparum antigen merozoite surface protein 119 reveals a protective role for invasion inhibitory antibodies. J Exp Med.

[pmed-0040320-b065] Cavanagh DR, Dodoo D, Hviid L, Kurtzhals JA, Theander TG (2004). Antibodies to the N-terminal block 2 of Plasmodium falciparum merozoite surface protein 1 are associated with protection against clinical malaria. Infect Immun.

[pmed-0040320-b066] al-Yaman F, Genton B, Kramer K, Chang S, Hui G (1996). Assessment of the role of naturally acquired antibody levels to Plasmodium falciparum merozoite surface protein-1 in protecting Papua New Guinean children from malaria morbidity. Am J Trop Med Hyg.

[pmed-0040320-b067] Egan AF, Morris J, Barnish G, Allen S, Greenwood BM (1996). Clinical immunity to Plasmodium falciparum malaria is associated with serum antibodies to the 19-kDa C-terminal fragment of the merozoite surface antigen, PfMSP-1. J Infect Dis.

[pmed-0040320-b068] John CC, O'Donnell RA, Sumba PO, Moormann AM, de Koning-Ward TF (2004). Evidence that invasion-inhibitory antibodies specific for the 19-kDa fragment of merozoite surface protein-1 (MSP-1 19) can play a protective role against blood-stage Plasmodium falciparum infection in individuals in a malaria endemic area of Africa. J Immunol.

[pmed-0040320-b069] Polley SD, Tetteh KK, Lloyd JM, Akpogheneta OJ, Greenwood BM (2007). Plasmodium falciparum merozoite surface protein 3 is a target of allele-specific immunity and alleles are maintained by natural selection. J Infect Dis.

[pmed-0040320-b070] Badell E, Oeuvray C, Moreno A, Soe S, van Rooijen N (2000). Human malaria in immunocompromised mice: an in vivo model to study defense mechanisms against Plasmodium falciparum. J Exp Med.

[pmed-0040320-b071] Druilhe P, Spertini F, Soesoe D, Corradin G, Mejia P (2005). A malaria vaccine that elicits in humans antibodies able to kill Plasmodium falciparum. PLoS Med.

[pmed-0040320-b072] Lundquist R, Nielsen LK, Jafarshad A, Soesoe D, Christensen LH (2006). Human recombinant antibodies against Plasmodium falciparum merozoite surface protein 3 cloned from peripheral blood leukocytes of individuals with immunity to malaria demonstrate antiparasitic properties. Infect Immun.

[pmed-0040320-b073] Jafarshad A, Dziegiel MH, Lundquist R, Nielsen LK, Singh S (2007). A novel antibody-dependent cellular cytotoxicity mechanism involved in defense against malaria requires costimulation of monocytes FcgammaRII and FcgammaRIII. J Immunol.

[pmed-0040320-b074] Hisaeda H, Saul A, Reece JJ, Kennedy MC, Long CA (2002). Merozoite surface protein 3 and protection against malaria in Aotus nancymai monkeys. J Infect Dis.

[pmed-0040320-b075] Druilhe P, Perignon JL (1997). A hypothesis about the chronicity of malaria infection. Parasitol Today.

[pmed-0040320-b076] Saul A (2007). Malaria vaccines based on the Plasmodium falciparum merozoite surface protein 3–should we avoid amino acid sequence polymorphisms or embrace them?. J Infect Dis.

[pmed-0040320-b077] Singh S, Soe S, Roussilhon C, Corradin G, Druilhe P (2005). Plasmodium falciparum merozoite surface protein 6 displays multiple targets for naturally occurring antibodies that mediate monocyte-dependent parasite killing. Infect Immun.

[pmed-0040320-b078] Mills KE, Pearce JA, Crabb BS, Cowman AF (2002). Truncation of merozoite surface protein 3 disrupts its trafficking and that of acidic-basic repeat protein to the surface of Plasmodium falciparum merozoites. Mol Microbiol.

[pmed-0040320-b079] Oeuvray C, Bouharoun-Tayoun H, Filgueira M-C, Gras-Masse H, Tartar A (1993). Characterization of a Plasmodium falciparum merozoite surface antigen targeted by defense mechanisms developped in immune individuals. CR Acad Sci Paris.

[pmed-0040320-b080] Audran R, Cachat M, Lurati F, Soe S, Leroy O (2005). Phase I malaria vaccine trial with a long synthetic peptide derived from the merozoite surface protein 3 antigen. Infect Immun.

